# Effectiveness of a Theory-Based Intervention in Improving Bacillus Calmette-Guérin Uptake and Preventive Practices of Childhood Tuberculosis Among Pregnant Women: Protocol for a Randomized Control Trial

**DOI:** 10.2196/68088

**Published:** 2026-02-09

**Authors:** Ahmed Mohamed Dirie, Nor Afiah Mohd Zulkefli, Salmiah Md Said, Ahmed Zaid Fatah Azman, Kassim Abdi Jimale, Osman Abubakar Fiidow

**Affiliations:** 1 Department of Community Health, Faculty of Medicine and Health Sciences, Universiti Putra Malaysia, Serdang, Selangor, 43400, Malaysia, 60 39769 2536; 2 Faculty of Medicine and Health Sciences, Jamhuriya University of Science and Technology, Mogadishu, Somalia

**Keywords:** childhood tuberculosis, prevention practices, Bacillus Calmette–Guérin, BCG, randomized controlled trial, information-motivation-behavioral (IMB) skills model

## Abstract

**Background:**

Tuberculosis (TB) in children is one of the most significant public health crises in Somalia. This issue is aggravated by the fact that only 36.7% of children aged 12 to 23 months receive the Bacillus Calmette–Guérin (BCG) vaccine in Somalia, which helps prevent TB-disseminated diseases. Among the major factors that contribute to poor BCG uptake and TB prevention practices are the lack of maternal knowledge, negative attitude, and poor self-efficacy toward the BCG vaccine and TB prevention practices. As such, pregnant women play a vital role in ensuring timely, routine BCG vaccination for their newborns and adherence to TB prevention practices.

**Objective:**

This study aims to develop, implement, and evaluate the impact of health education intervention using the information-motivation-behavioral (IMB) skills model to improve knowledge, attitudes, and self-efficacy regarding BCG uptake and TB prevention among pregnant women in Banadir Hospital.

**Methods:**

This single-blind randomized controlled trial enrolled a sample of 370 pregnant women recruited at Banadir Hospital in Mogadishu, Somalia. Eligible participants will be randomized to an intervention group receiving an IMB-based health education and to a waiting list control group, in a 1:1 ratio. Outcome assessments will be conducted at baseline, 2-month follow-up, and 4-month follow-up. The primary outcomes are BCG vaccine uptake and TB prevention practices. Secondary outcomes include knowledge, attitudes, and self-efficacy related to the BCG vaccine and TB prevention practices. The IMB-based health education intervention program consists of 6 sessions, with 1 group per session, and with each session containing 30 participants. The effects of the intervention will be assessed by handing out the same self-administered questionnaires at baseline, 2-month postintervention, and 4-month postintervention.

**Results:**

A total of 370 pregnant women were recruited at baseline in November 2021, with 185 assigned to the intervention group and 185 assigned to the control group. In January 2022, the 185 pregnant women enrolled in the intervention program and completed the sessions by February 2022. Data collection for the 2-month and 4-month postintervention assessments was completed in June 2022. The findings of this study will be reported by the beginning of 2026.

**Conclusions:**

The developed health education intervention module in this study has the potential to be adopted and included as part of routine antenatal care services. Its implementation could effectively raise awareness among pregnant women in Somalia regarding the importance of BCG vaccination and TB prevention practices, ultimately mitigating childhood mortality rates associated with TB-disseminated diseases in the country.

## Introduction

Tuberculosis (TB) remains a prevalent health issue worldwide, with over 10 million new cases annually [1]. TB cases are also reported in children younger than 15 years and are referred to as childhood TB. Moreover, children younger than 5 years are particularly vulnerable to developing active TB disease [2]. In 2011, the World Health Organization reported its first estimate of annual global childhood TB, which amounted to 490,000 cases [3]. In 2022, approximately 1.25 million children and young adolescents were diagnosed with TB, with 47% of these cases occurring in children younger than 5 years. This age group represents 12% of the overall TB incidence of 10.6 million [4]. However, approximately 51% of these cases were either undiagnosed or not reported to national TB programs. The gap is even more pronounced among children younger than 5 years, with 58% of estimated cases being missed, compared to 30% for individuals aged 15 years and older [3].

The risk factors contributing to the development of TB disease among children have received increasing attention over the years. Contact with known adult TB cases is a significant risk factor for both children and adults [5,6]. A study conducted in Guinea-Bissau found that children younger than five years living in the same household as an adult with TB faced a markedly higher risk of death, with the risk being up to 8 times higher for those whose mothers were affected by the disease [7]. Other important risk factors include lack of BCG vaccination, severe malnutrition, and knowledge and attitude toward TB [8-10].

Somalia is classified as one of the least developed countries and faces a significant burden of TB, particularly multidrug-resistant TB [11]. In 2023, the TB incidence in Somalia was reported at 246 cases per 100,000 population [12]. Despite the heightened vulnerability of children due to malnutrition and limited access to health care, only about 56.4% of TB cases are detected, resulting in many children going without the necessary treatment [13]. Furthermore, according to the Somali National Strategic Plan 2020‐2024 for Tuberculosis, 22% of TB cases occur in children younger than 15 years [14].

Somalia, as a TB-endemic country, faces significant challenges, particularly low BCG vaccine uptake, limited knowledge of childhood TB, and negative attitudes toward the disease. According to the Somali Health Demographic Survey conducted in 2020, only 36.7% of children aged 12 to 23 months received the BCG vaccine, marking one of the lowest coverage rates in Africa [15]. Similarly, community-based studies in Somalia revealed that only 4.7% of participants correctly identified bacteria as the cause of TB, while just 44.7% of participants were aware of the importance of covering their nose and mouth when coughing and sneezing to prevent TB transmission [16,17]

Previous intervention studies have demonstrated the positive impact of health education on improving BCG uptake and TB prevention practices [18,19]. Similarly, health theories are important for understanding health behaviors, designing effective interventions, and evaluating program success. This study suggests using the information-motivation-behavioral (IMB) skills model construct over theories such as the health belief model and the theory of planned behavior, which predominantly center on perceptions and beliefs concerning health threats but may underestimate the crucial motivational aspect in behavior change. The IMB skills model emphasizes 3 essential constructs for promoting behavior change: providing relevant information regarding the desired behavior (which alone is insufficient for behavior change), fostering appropriate motivation to engage in the desired behaviors, and ensuring that individuals possess the necessary skills and competencies to effectively perform the desired behaviors [20].

However, no studies were identified during the literature search that assessed health education interventions specifically related to a model concerning BCG vaccination and TB prevention in Somalia. Therefore, this study proposes to develop, implement, and assess the effects of an IMB-based health education intervention on BCG uptake and TB prevention practices among pregnant women attending the antenatal care (ANC) clinic at Banadir Maternal and Child Hospital, Mogadishu, Somalia.

## Methods

### Study Location

This study selected Banadir Maternal and Child Hospital, which is a public referral hospital, as the study location. This hospital is located in Mogadishu, the capital city of Somalia. Also known as Xamar, it is the most populous city in Somalia, with an estimated 2 million residents [21], and stretches along the southern coast of Somalia on the Horn of Africa. This particular hospital was chosen due to its strategic geographical location at the center of the city and its considerable size and high patient load, making it one of the largest hospitals in the area. The hospital comprises 2 main departments: maternity and pediatrics. Under the Maternity Department, the hospital operates an ANC clinic from Saturday to Thursday, providing support to expectant mothers throughout their pregnancy. Other essential services that are available at this hospital include the Diarrhea Treatment Center, Blood Transfusion Unit, Emergency Unit, TB Center, and Research and Training Center.

### Study Design

A single-blind randomized controlled trial will be conducted in this study, with only the participants blinded to their assigned group. The participants will be randomly categorized into the intervention group and control group. The study protocol will be reported in accordance with the Standard Protocol Items: Recommendations for Intervention Trial (SPIRIT) 2013 Guideline [22] (Figure S1, Multimedia Appendix 1).

### Study Duration

Planning of the study protocol began in January 2021, and data collection was completed in June 2022.

### Study Population and Study Setting

The study population consists of pregnant women attending the Banadir Maternal and Child Hospital, Mogadishu, for ANC visits. The inclusion criteria include mothers with a gestational age range of 25 to 33 weeks, plans for postnatal follow-up at the hospital, and residence in Mogadishu, including internally displaced persons. The exclusion criteria include mothers who visited the hospital for other health conditions, those of non-Somali nationality, and those who had previous health education training related to immunization and TB prevention practices.

### Sample Size

The 2-sample mean formula was used to determine the sample size for this study. This formula considers the mean and SD of TB preventive practices from a previous health education program conducted by John [19], where a SD of 4.40, an intervention group mean of 20.71, and a control group mean of 18.45 were used in the calculation. To ensure adequate statistical power, several adjustments were made for the estimated eligibility (90%) and attrition rate (30%), with a 95% significance level and 80% desired power. The anticipated high attrition rate is primarily due to the impact of the COVID-19 pandemic, which created significant barriers to participation in the study. Based on this formulation, the total sample size for this study was 370 participants, with an equal number of participants in both the intervention and control groups.

### Recruitment

Potential participants were recruited over a 4-week period from the ANC clinic at Banadir Maternal and Child Hospital. To ensure an organized and systematic selection, their names were sorted into the ANC electronic register in chronological order. After obtaining informed consent from all participants who agreed to take part in the study, their eligibility was assessed by applying the inclusion and exclusion criteria checklist. The recruitment process is summarized in Figure S2 (Multimedia Appendix 1).

### Randomization, Allocation Concealment, and Blinding

Randomization was performed at the individual level, with pregnant women as the sampling unit. Ensuring an equal allocation ratio (1:1), participants were randomly assigned into the intervention group, which received the IMB-based health education intervention, or the control group, which was placed on a waiting list. The randomization process was conducted using computer-generated random numbers using a web-based calculator (GraphPad; GraphPad Software) by hospital staff who were independent of all other research processes. The generated sequences were placed in sealed opaque envelopes. Subsequently, another hospital staff member, who was also independent of the research team, was responsible for opening the envelopes sequentially and assigning participants to either the intervention or control group.

### Intervention

#### Intervention Development and Validation

The intervention modules were developed based on relevant sources from the World Health Organization, the Ministry of Health of Somalia [23], and publications and guidelines from previous studies [24]. As presented in Table 1, the IMB components, namely information, motivation, and behavior skills, were used to develop the intervention program. Each module’s content was examined by a panel of experts consisting of 6 public health specialists and pediatricians from Universiti Putra Malaysia, the Ministry of Health of Somalia, and Bandir Hospital. To ensure effective delivery of the module content to participants, 2 female intern students provided a single, intensive training session lasting approximately 3 hours. To further prepare the interns, a follow-up session was scheduled a day before the intervention sessions began. Both interns had backgrounds in medicine and have demonstrated competency in Somali communication, which enhanced their ability to engage effectively with participants.

**Table 1.** Summary of intervention program content and delivery.

Theory construct module	Health education content	Delivery strategy	Estimated duration
Module 1: information	Basic knowledge of childhood TB^a^, including cause, transmission, signs and symptoms, severity, and complications.	Lecture using PowerPoint presentation, question-and-answer sessions.	30 min
Module 2: information	Benefits, schedule, and side effects of the BCG^b^ vaccine; respiratory hygiene (cough etiquette); and environmental control measures.	Lectures using PowerPoint presentations, question-and-answer sessions.	30 min
Module 3: motivation	Importance of the BCG vaccine, respiratory hygiene (cough etiquette), and environmental control measures; religious and cultural leaders addressing the benefits, safety, and halal status of the BCG vaccine, and normalizing face mask use.	Interactive discussion, brainstorming sessions, video presentations, and lectures.	1 h
Module 4: behaviorSkills	Self-efficacy evaluation related to receiving the BCG vaccine and childhood TB prevention practices; problem-solving focused on challenges and solutions; reinforcement through a 1-month follow-up call for mothers in the intervention group to reinforce key intervention points and prioritize the administration of the BCG vaccine for their newborns.	Role play (video), interactive session, brainstorming exercise, and a 1-month follow-up call to reinforce.	1 h and 30 min

a TB: tuberculosis.

b BCG: Bacillus Calmette–Guérin.

Pretesting of the questionnaire was conducted among 36 pregnant women in Banadir Hospital who were not part of the study to assess the reliability of the questionnaire. Reliability was carried out using Cronbach α to assess for internal consistency. The Cronbach α coefficients for knowledge, attitudes, and prevention practices were 0.774, 0.829, and 0.689, respectively. The results were within the acceptable range of 0.60 to 0.95 [25].

#### Intervention Structure and Delivery

The intervention consists of 4 modules. The first module utilizes a combination of lectures, PowerPoint presentations, and a question-and-answer session to provide a foundational understanding of childhood TB. The duration of this module is estimated to be 30 minutes. The second module concentrates on understanding the BCG vaccine and TB prevention practices. The delivery method is similar to that of the first module and takes approximately 30 minutes. The third module explores participants’ attitudes toward the BCG vaccine and TB prevention practices. Various strategies are used, including video presentations featuring traditional and religious leaders, interactive sessions, and brainstorming exercises. This module is estimated to take 1 hour to be completed. The fourth and final module aims to enhance participants’ self-efficacy regarding the BCG vaccine and TB prevention practices, as well as equip participants with useful skills to make informed decisions and overcome potential challenges. This module incorporates a diverse range of strategies, including role-play videos, interactive sessions, and brainstorming exercises. The duration of this module is estimated to last 1 hour and 30 minutes.

Participants in the intervention group were divided into 6 small groups for health education sessions. There were 6 intervention sessions, with 1 group per session; each session accommodated 30 participants, except for the first session, which had 35 participants. The language of delivery was Somali, and participants received US $2.00 for transportation. The delivery of the modules was closely monitored and supervised by the researchers, who provided necessary feedback to the facilitators to ensure strict adherence to the protocol without deviation. After the completion of the sessions, mothers participating in the intervention received a follow-up call 1 month later to reinforce the key points discussed and to highlight the importance of prioritizing the administration of the BCG vaccine for their newborns.

#### Control Group

The control group was assigned to a waiting list status throughout the intervention program. Participants received their usual care during their visits to the ANC clinic and had access to general health education content through television programming. The hospital does not provide personalized health education for antenatal mothers. To avoid potential contamination or cross-group influence, participants in both groups were given different follow-up dates. Additionally, participants were clearly instructed on the importance of not discussing the intervention sessions with others.

### Data Collection

Data were collected using paper questionnaires at 3 distinct time intervals: baseline, 2-months postintervention, and 4-months postintervention. Participants were asked to complete a consent form before participating and were informed that their information would remain confidential and used solely for research purposes. The recorded data were securely stored in protected files, and individual responses were not reported publicly; instead, findings were presented in aggregate form. The random assignment took place on the same day that participants were selected for the study, after the collection of baseline data. To facilitate the data collection process, each participant was given a study follow-up card and was requested to carry them during each follow-up visit to the hospital.

### Quality Control

Several steps were taken to ensure the validity and reliability of the questionnaire. First, content and face validity were evaluated to ensure that the wording, ordering, and language were appropriate and well understood. The analysis categorized items as good, average, or poor; language clarity was assessed as clear, average, or confusing; and the appropriateness of construct measurement was classified as good, average, or poor. Feedback from experts and participants led to important modifications, including rewording several items for clarity and removing 3 items deemed irrelevant to the study objectives.

Additionally, factor analysis was conducted using SPSS software (version 27.0; IBM Corp) on a separate sample set of 134 participants who were not part of the main study but were recruited from the same hospital to assess sampling adequacy. Cohen’s κ and intraclass correlation coefficient values were also utilized to evaluate the reliability of the questionnaire. All values exceeded 0.60, which is considered acceptable [25].

### Outcome Assessment

Table 2 presents the primary and secondary outcome assessments, including the timing of each measurement.

**Table 2.** Outcome measurements.

Outcome measure	Definition	Time of measurement		
		T0 (baseline)	T1 (2-month follow-up)	T2 (4-month follow-up)
Primary outcomes				
BCG^a^ uptake	Refers to receiving 1 dose of the BCG vaccine at birth. Infants who receive the vaccine after 6 weeks will be considered delayed.	No	Yes	Yes
TB^b^ preventive practices	Refers to the change in total practice scores of actions taken by participants to reduce the risk of TB infection and transmission, including respiratory hygiene (cough etiquette) and environmental control, between baseline, 2-month follow-up, and 4-month follow-up.	Yes	Yes	Yes
Secondary outcomes				
Knowledge	Refers to the total score change of participants’ knowledge regarding BCG vaccine benefit, schedule, and side effect, as well as knowledge of preventive practices (respiratory hygiene/cough etiquette and environmental control) between baseline, 2-month follow-up, and 4-month follow-up.	Yes	Yes	Yes
Attitude	Refers to the change in total attitude scores toward the BCG vaccine and preventive practices between baseline, 2-month follow-up, and 4-month follow-up.	Yes	Yes	Yes
Self-efficacy	Refers to the change in total scores reflecting participants’ confidence in their ability to obtain and receive the BCG vaccine, as well as their confidence in engaging in TB prevention practices between baseline, 2-month follow-up, and 4-month follow-up.	Yes	Yes	Yes

a BCG: Bacillus Calmette–Guérin.

b TB: tuberculosis.

### Data Analysis

Collected data were entered into SPSS software (version 27.0; IBM Corp) before proceeding with data cleaning procedures. Both statistical and graphical normality tests were applied to continuous variables, such as age, knowledge, attitude, and self-efficacy. At baseline, the comparison of variables between groups used appropriate statistical measures based on the distribution of data. For normally distributed data, results are reported as mean (SD), and comparisons were conducted using an independent *t* test. For non-normally distributed data, results were reported as median (IQR), and comparisons were conducted using the chi-square test and the Mann-Whitney *U* test.

To evaluate the main effect of the IMB-based intervention modules on the response variables over time, as well as the group-time interactions, repeated-measures ANOVA was used for normally distributed data. For non-normally distributed data, generalized linear mixed models were applied to account for individual-level variability among participants, or generalized estimating equations were used when focusing on overall population-level trends. Intention-to-treat analyses were conducted separately for the intervention and control groups, and missing data were addressed using multiple imputations. Results were reported as differences between groups with 95% CIs. Statistical significance in this study was set at α=.05.

### Ethical Considerations

The trial protocol was approved by the Ethics Committee for Research Involving Human Subjects at the Universiti Putra Malaysia (UPM) (UPM/TNCPI/RMC/JKEUPM/1.4.18.2), the Ethical Review Committee of the Ministry of Health of Somalia (MOHHS/DGO/1166/Feb2024), and Banadir Hospital (BH/30594/4/22). The study was registered on May 24, 2022, with the Pan African Clinical Trials Registry and assigned a unique identification number PACTR202105554130782.

A participant information sheet in the Somali language was provided in written form by trained enumerators to each participant, from whom informed consent was obtained. Participant information and identities remained confidential and used solely for the purpose of this research. Participation in the study was voluntary, and participants had the right to withdraw at any point during the intervention. Participants received compensation of USD 2.00 per follow-up visit to cover transportation costs.

Any significant protocol modifications, such as alterations to eligibility criteria, outcomes, or analysis methods, will be promptly communicated to both the Pan African Clinical Trials Registry and the Jawatankuasa Etika Universiti Putra Malaysia.

## Results

Recruitment began on November 25, 2021, and by December 21, 2021, a total of 370 participants had been enrolled in the trial. The intervention sessions were completed on February 4, 2022. Follow-up data collection was completed on June 14, 2022. Results are expected to be published by the end of 2025.

## Discussion

### Expected Findings and Implications

Health theories play a crucial role in public health by providing frameworks for understanding health behaviors, designing effective interventions, and evaluating improvements of outcomes. This study will use IMB-based theory constructs to improve knowledge, attitudes, and self-efficacy among antenatal mothers, ultimately aiming to improve BCG uptake and childhood TB prevention practices. However, previous studies in Somalia have primarily been observational in design and have lacked a theoretical framework, which limits an inclusive understanding of the factors influencing behavior change. Therefore, investigating this gap is essential for improving outcomes for mothers and children.

This study is the first to examine the effectiveness of an IMB-based health education intervention in improving BCG uptake and childhood TB prevention practices by comparing intervention and control groups. In particular, the findings of this study may highlight the potential for improving maternal knowledge, attitudes, and self-efficacy regarding BCG vaccination and childhood TB prevention practices in Mogadishu, Somalia. Similarly, it is expected that pregnant women in the intervention group will demonstrate higher TB prevention practices and increased BCG uptake for their children compared with those in the control group. Previous intervention studies supported the effectiveness of health education interventions in improving BCG uptake and TB prevention practices [26-28]. Finally, the findings are anticipated to contribute to improving TB prevention efforts in Somalia, where low BCG coverage, poor maternal knowledge, and fragile health systems continue to pose major public health challenges.

### Strengths and Limitations

This is the first IMB-based health education intervention designed to improve BCG uptake and childhood TB prevention practices. Additionally, its randomized controlled design, single-blind allocation, theoretical framework, and use of validated tools enhance internal validity and reduce the risk of bias. Furthermore, conducting the trial at Banadir Hospital, a referral facility for maternal and child health, allows for the recruitment of a diverse population of pregnant women, including internally displaced persons, thereby enhancing the generalizability of outcomes.

Nevertheless, this study has several limitations. Despite blinding, cross-group information sharing among participants cannot be entirely ruled out, which may influence the findings. Additionally, outcomes are largely based on self-reported measures, introducing the potential for reporting bias. The inability to blind outcome assessors may also introduce bias in the evaluation of findings. Furthermore, because the trial is facility-based, the findings may not be fully generalizable to women who lack access to health care. Finally, external factors, such as sociopolitical instability or disruptions in the health system, may independently affect BCG uptake regardless of the intervention.

### Conclusion

This study is a single-blinded randomized controlled trial involving 370 mothers at Banadir Hospital in Mogadishu, Somalia. Participants were randomized into either an intervention group receiving the IMB-based health education intervention or a waitlist control group. It is anticipated that pregnant women in the intervention program will show significant improvements in the primary outcomes of BCG vaccine uptake and TB prevention practices. Additionally, secondary outcomes, including knowledge, attitudes, and self-efficacy related to childhood TB prevention and the BCG vaccine, are expected to improve compared with those in the control group.

The effectiveness of the IMB-based health education modules developed in this intervention may have the potential to be adopted in routine health education programs for pregnant women during their ANC visits in Mogadishu, Somalia.

## Supplementary material

10.2196/68088 Multimedia Appendix 1 Figures S1-S2

## Abbreviations

ANC: antenatal
BCG: Bacillus Calmette-Guérin
IMB: information-motivation-behavior
RCT: randomized controlled trial
TB: tuberculosis
