# Supplementary material for: Effectiveness of a Theory-Based Intervention in Improving Bacillus Calmette-Guérin Uptake and Preventive Practices of Childhood Tuberculosis Among Pregnant Women: Protocol for a Randomized Control Trial
Source: JMIR Res Protoc. 2026 Feb 9;15:e68088. doi: 10.2196/68088 (PMC12887941; doi:10.2196/68088)
Supplement: Multimedia Appendix 1 [file resprot-v15-e68088-s001.docx]

|  | **Study Period** | | | | | |
| --- | --- | --- | --- | --- | --- | --- |
|  | **Enrolment** | **Allocation** | **Post allocation** | | | **Close-out** |
| **TIMEPOINT**** | ***11/2021*** | **12/2021** | **1/2022** | **3/2022** | **5/2022** | **5/2022** |
| **ENROLMENT:** |  |  |  |  |  |  |
| **Eligibility screen** | X |  |  |  |  |  |
| **Informed consent** | X |  |  |  |  |  |
| **Allocation** |  | X |  |  |  |  |
| **INTERVENTION**  **IMB-based health education** |  |  | X |  |  |  |
| **ASSESSMENTS:** |  |  |  |  |  |  |
| ***BASELINE*** | X |  |  |  |  |  |
| ***Primary outcome*** |  |  |  | X | X |  |
| ***Secondary outcome*** |  |  |  | X | X |  |
| ***Endpoint outcomes*** |  |  |  |  |  | X |

Figure S1: Standard Protocol Items Recommendations for Interventional Trials (SPIRIT) 2013 guideline. Schedule of enrollment, interventions, and assessment.

**Analysis:**

Per-protocol (n = ?)

Intention-to-treat (n = ?)

**Analysis:**

Per-protocol (n = ?)

Intention-to-treat (n = ?)

**4-month follow-up**

**2-month follow-up**

**2-month follow-up**

**4-month follow-up**

Allocated to intervention group (n = 185)

Assessed for eligibility

(n = 468)

(n= 468)

Excluded (n = 98)

- Not meeting the inclusion criteria (n = 62 in the first trimester)
- Decline to participate (n = 36)

Randomised (n = 370)

Allocated to control group

(n = 185)

Enrollment

Allocation

Follow-up

Analysis

Figure S2: Consort study flow chart of the intervention and control groups.
